# Psychometric evaluation of the factor structure of the General Health Questionnaire-12 in a sample of Chinese university students

**DOI:** 10.3389/fpubh.2026.1849577

**Published:** 2026-07-28

**Authors:** Sai-fu Fung, Cyrus Ngo Tin Lai, Jerf W. K. Yeung

**Affiliations:** 1 Department of Social and Behavioural Science, City University of Hong Kong, Kowloon, Hong Kong SAR, China; 2 Jockey Club School of Public Health and Primary Care, The Chinese University of Hong Kong, New Territories, Hong Kong SAR, China

**Keywords:** CFA, Chinese, General Health Questionnaire, GHQ-12, psychological distress, student

## Abstract

**Aims:**

The 12-item General Health Questionnaire (GHQ-12) is widely used for assessing psychological distress, yet debates persist regarding its optimal factor structure, especially across different cultural contexts and demographic groups. While the bi-factor model has gained empirical support in Western populations, limited research has examined its validity in Chinese university students. This study aimed to evaluate the psychometric properties of the GHQ-12 in Chinese university students by examining its factor structure through Classical Test Theory.

**Methods:**

A cross-sectional study was conducted with a sample of Chinese university students (*n* = 511). The dataset was randomly split into two samples, sample 1 (*n* = 260) and sample 2 (*n* = 251) for EFA and CFA, respectively. The study evaluated the construct validity of the GHQ-12 one-factor, two-factor, three-factor, and bi-factor models using CFA.

**Results:**

EFA revealed a two-factor structure with strong fit. CFA results demonstrated that the bi-factor model showed the best overall numerical fit, whereas the two-factor model was retained as the preferred solution because it demonstrated strong and balanced factor loadings, conceptual interpretability, and greater parsimony (*χ*^2^ = 83.3, df = 53; CFI = 0.989; TLI = 0.986; RMSEA = 0.048; SRMR = 0.063). Internal consistency was excellent (*α* = 0.838, *ω* = 0.842), and convergent validity was moderate with WHO-5 (*r* = −0.339) and SWEMWBS (*r* = −0.475).

**Conclusion:**

The GHQ-12 demonstrates acceptable psychometric properties in Chinese university students. The two-factor model was retained as the preferred solution based on interpretability and parsimony, although the bi-factor model showed superior global fit indices. These findings support the use of the GHQ-12 for mental health screening and future psychometric research in Chinese youth populations.

## 1 Introduction

Mental health has emerged as one of the most pressing global public health challenges of the 21st century. According to the World Health Organization (WHO), approximately one in eight people worldwide lives with a mental disorder, with anxiety and depressive disorders being the most prevalent (1). Psychological distress, broadly defined as a state of emotional suffering characterized by symptoms of depression, anxiety, somatic complaints, and impaired functioning in response to internal or external stressors (2, 3), is particularly salient during the transition period from adolescence to emerging adulthood. It is suggested that understanding psychological distress between adolescence and emerging adulthood would give valuable insight for prevention (4). Among different questionnaires, the General Health Questionnaire (GHQ) is widely recommended tool, used self-report instruments for detecting non-psychotic psychiatric disorders, particularly psychological distress in community and primary care settings (5). The GHQ is a multidimensional tool to assess recent changes related to mental health, such as anxiety, depression, social dysfunction, and loss of confidence (6, 7). While psychological distress has sometimes been treated as essentially unidimensional for practical screening purposes, theoretical and empirical work suggests it is inherently multidimensional, encompassing affective, cognitive, and social domains (7, 8). These differing conceptualizations have led to ongoing debates regarding the optimal factor structure of distress measures. Among different versions, GHQ-12 is one of the commonly adopted versions (9). The scale has been translated into over 40 languages and validated in numerous cultural contexts (9, 10). Meta-analyses have consistently reported good internal consistency and acceptable validity (5, 10).

The GHQ-12 has been extensively applied in diverse research and clinical contexts beyond initial screening. In community-based studies, it has tracked psychological distress trends in general populations, such as during pandemics and in migrant health programs (11, 12). In intervention research, it serves as a primary outcome measure in randomized controlled trials evaluating mindfulness programs (13, 14), cognitive-behavioural therapies (15), and workplace wellness initiatives (16), demonstrating sensitivity to change pre- and post-intervention. Its brevity and multidimensionality make it ideal for longitudinal studies and cross-cultural comparisons in mental health promotion.

The GHQ-12 offers distinct advantages for university screening contexts. Its brevity (12 items) makes it feasible for routine administration in student health services, where longer instruments such as the 90-item Symptom Checklist-90-R (SCL-90-R) are less practical for broad screening programs (17). Unlike the Patient Health Questionnaire-9 (PHQ-9) (18), which focuses specifically on depression, the GHQ-12 was designed to screen for non-specific psychological distress and includes items assessing self-confidence (7, 8). Furthermore, the GHQ-12 has established normative data across diverse populations, facilitating cross-cultural and cross-study comparisons. While the PHQ-9 has been validated for Chinese student populations (19), its focus on a single symptom cluster limits its utility for comprehensive mental health screening. The GHQ-12’s comprehensive yet efficient assessment of general psychological distress, combined with its established cross-cultural validity in Chinese populations (20, 21), makes it particularly suitable for university mental health screening programs.

However, debates continue to emerge regarding its factor structure (22–24). For example, the two-factor model has been proposed by Andrich and van Schoubroeck (25) to distinguish positive and negative wording effects (e.g., positively phrased items like “felt happy” vs. negatively phrased ones like “felt unhappy”). The three-factor model suggests delineating anxiety/depression, social dysfunction, and loss of confidence (8). Originally conceptualized as a unidimensional scale, the GHQ-12 has shown considerable variability in its factor structure across populations (6, 7). Recently, the bi-factor model has gained attention for reconciling these perspectives by accounting for method artifacts such as response biases (26). In the bi-factor model, all items load on a general psychological distress factor that captures the shared common variance, while orthogonal specific factors (usually positive and negative wording factors) account for residual variance attributable primarily to method effects rather than distinct substantive dimensions (10, 26). This approach is theoretically valuable as it simultaneously supports the use of a total score while acknowledging multidimensionality and wording effects. Gnambs and Staufenbiel (10) conducted a meta-analytic structural equation modelling (MASEM) study on this debate. They argued that separate subscale scores (e.g., positive vs. negative items, or three-factor solutions like “anxiety,” “social dysfunction,” “loss of confidence”) are not recommended because they mostly reflect the general factor and have low unique reliability and limited incremental validity. This has opened up recent discussions on the bi-factor structure of the GHQ-12, with various studies advocating and validating bi-factor models with better construct validity (24, 27, 28). However, many studies still suggest otherwise, advocating for two or three-factor models of the GHQ-12 (21, 29–32). This indicates a need to revisit the dimensionality and factor structure of GHQ-12.

Observing these unaligned results, the existing literature argued that these inconsistencies are particularly salient in non-Western contexts, where cultural response styles (e.g., modesty or indirect expression) may amplify wording effects (20). In China, university students navigating intense academic competition, family expectations, and rapid societal change, represent a high-risk group for psychological distress (4). Yet prior Chinese validations have largely targeted rural residents (21), pregnant women (30), older adult populations (31), or professionals (32, 33), leaving a gap in research on Chinese mainland university students. The present study addresses this by re-evaluating the dimensionality and factor structure of the GHQ-12 in a sample of Chinese university students.

## 2 Methods

### 2.1 Participants

A total of 511 participants (85.5% female; *M* = 20.41, SD = 2.49) from China were recruited for this study, which focused on university students in the university context. Data collection took place in April and May 2019 via the university’s student intranet system, where the survey link was posted and made available to all students in the university. Students who clicked the link, read the study information, and provided informed consent were enrolled in the study.

The inclusion criteria were: (1) being a university student, (2) being able to understand Chinese, and (3) providing informed consent to participate voluntarily. The exclusion criteria were: (1) inability to understand Chinese and (2) refusal to provide consent. The dataset was collected specifically for the purpose of validating the GHQ-12 and was not derived from any existing institutional screening process. Based on an estimated university population of around 20,000 students, the response rate was approximately 2.56%.

No missing data were observed because the online survey required participants to complete all items before submission. The recruited sample reflected the gender ratio profile of that university. The high proportion of female participants reflected the gender distribution of the participating university; however, this imbalance may limit the generalizability of the findings. With a sample size of 511, the estimated margin of error was approximately ±4.28% at the 95% confidence level. To ensure confidentiality, all recorded information was anonymised and contained no identifying details. Participation was voluntary, with informed consent obtained beforehand, and participants could withdraw from the study at any time during data collection. The study received approval from the university’s research ethics committee and adhered fully to internationally recognised ethical principles, including the Declaration of Helsinki, as well as relevant national rules and regulations.

### 2.2 Measures

Following the dual-continuum model of mental health (34), which posits that mental wellbeing and psychological distress are related but distinct constructs, the WHO-5 and SWEMWBS were selected to examine the convergent validity of the GHQ-12 via expected negative associations with wellbeing. Although these wellbeing measures provide valuable evidence of convergent validity, we acknowledge that this study did not include more direct indicators of psychological distress or psychopathology (e.g., measures of anxiety or depression symptoms), which would have strengthened convergent validity evidence.

The GHQ-12 (6, 7) is a widely used self-report screening tool for detecting non-specific psychological distress. It consists of 12 items assessing symptoms over the past few weeks, with six positively worded (e.g., “Been able to concentrate on whatever you are doing”) and six negatively worded (e.g., “Lost much sleep over worry”). Responses are rated on a 4-point Likert scale (0 = not at all, 3 = much more than usual for positive items; reverse-scored for negative items). Total scores range from 0 to 36, with higher scores indicating greater distress. In the present study, the Likert (0–3) scoring method was used for all statistical analyses, including EFA, CFA, reliability estimation, and convergent validity correlations. The GHQ-12 has demonstrated strong psychometric properties across diverse populations (9). The Chinese version, validated previously (20), was used; items 2, 5, 6, 9, 10, and 11 were reverse-scored as needed.

The World Health Organization–Five Well-Being Index (WHO-5) is a self-reported measure consisting of five items, using a six-point Likert-type scale ranging from 0 (at no time) to 5 (all of the time). The sample questions like: “I have felt cheerful and in good spirits” and “My daily life has been filled with things that interest me.” Higher scores indicate a greater level of wellbeing (35, 36). The Chinese version was validated by Fung, Kong (37), Cronbach’s alpha is 0.905.

The Short Warwick Edinburgh Mental Well-Being Scale (SWEMWBS) is another self-reported measure comprising of seven positively worded items to assess individual hedonic and eudaimonic wellbeing (38, 39). Sample questions include: “I’ve been feeling useful” and “I’ve been feeling close to other people.” Each item is rated on a five-point scale, ranging from 1 (none of the time) to 5 (all of the time). The Chinese version of SWEMWBS was validated recently (40), Cronbach’s alpha is 0.883.

### 2.3 Data analysis

Analyses were conducted using SPSS and R (version 4.3.1). We randomly split the sample (*n* = 511) into two sets, sample 1 (*n* = 260) and sample 2 (*n* = 251), to avoid the potential problem of overfitting by running EFA and CFA on the same dataset (41). This sequential approach was chosen for several methodological reasons. First, although the GHQ-12 has established factor structures in the literature, its structure in Chinese university students—a group facing distinct developmental and academic stressors—has not been systematically examined. Given that previous studies have reported inconsistent factor structures in Chinese populations (e.g., two-factor vs. three-factor vs. bi-factor) (21, 30, 31), imposing a single theoretical model without preliminary exploration could risk misspecification. Second, conducting EFA on sample 1 allowed us to explore the emergent factor structure without imposing *a priori* constraints, which is particularly important when examining established scales in new populations (42). Third, the EFA results guided our selection of CFA models to test on the independent sample 2, serving as a cross-validation approach. This split-sample EFA-CFA design aligns with prior studies (31, 32, 43).

The random split was conducted using SPSS, assigning each participant to one of the two subsamples without replacement. Sample 1 was used to perform descriptive statistics and EFA, while sample 2 was used to conduct CFA and evaluate convergent validity of the GHQ-12. Item descriptive statistics, skewness, kurtosis, corrected item-total correlations, and Cronbach’s alpha (if item deleted) were computed using the psych package. McDonald’s omega was estimated for hierarchical reliability (44, 45). Convergent validity of GHQ-12 was evaluated with scales, including WHO-5 (46–48) and SWEMWBS (49, 50). According to the above literature, we hypothesized that the GHQ-12 holds significant negative relationship with WHO-5 and SWEMWBS.

EFA was conducted in SPSS using maximum likelihood extraction with oblique (direct oblimin) rotation. Oblique rotation was chosen because the underlying factors were theoretically expected to correlate, as is common in psychological scales (51, 52). Factorability was examined using the Kaiser–Meyer–Olkin (KMO) measure and Bartlett’s test of sphericity (53).

CFA was conducted to examine the factor structure of the GHQ-12 in China. We tested one-, two-, three-factor, and bi-factor models by using the lavaan package (54). Items were treated as ordered categorical variables, with estimation via weighted least squares mean- and variance-adjusted (WLSMV) (55). Fit was evaluated using *χ*^2^, RMSEA, comparative fit index (CFI), TLI, and standardized root mean square residual (SRMR). Acceptable fit criteria were RMSEA < 0.08, CFI/TLI > 0.95, SRMR < 0.08 (56).

To assess the potential influence of common method bias due to the self-report, single-source nature of the data collection, Harman’s single-factor test was conducted. All items from the GHQ-12, WHO-5, and SWEMWBS were entered into an unrotated principal component analysis. The first factor accounted for only 8.3% of the total variance, which is substantially below the commonly cited 50% threshold. This pattern did not suggest substantial common method variance or a dominant single-method factor in the present study (57). This result suggests that common method bias is not a major concern in the present study. In addition, no missing data treatment was required because the online survey was configured to require complete responses before submission.

## 3 Results

### 3.1 Descriptive statistics and internal consistency of GHQ-12 items

Descriptive statistics for the 12 GHQ items are presented in Table 1. Item means ranged from 1.3 (SD = 0.93) for GHQ5 to 2.3 (SD = 0.64–0.73) for GHQ4, GHQ7, GHQ8, and GHQ12, reflecting slightly higher endorsement of positively worded items relative to negatively worded ones. Skewness values ranged from −1.02 to 0.30, and kurtosis from −1.15 to 1.26, indicating mild deviations from normality that are acceptable for factor analytic procedures (58). Corrected item–total correlations were moderate to high, ranging from 0.293 (GHQ3) to 0.653 (GHQ10), suggesting good item discrimination and internal homogeneity. Although some researchers apply a strict threshold of 0.30 for item-total correlations (59), this criterion is somewhat arbitrary and depends on sample characteristics and scale length. We followed the more flexible convention that item-total correlations above 0.20 are acceptable for established scales examined in new populations, particularly when sample sizes exceed 200 (60). GHQ3’s corrected item-total correlation of 0.293 falls within this acceptable range, and its removal did not substantially improve Cronbach’s alpha (*α*iid = 0.839 vs. total *α* = 0.838). Furthermore, GHQ3 demonstrated satisfactory factor loadings in both EFA (0.418) and CFA (0.512), supporting its retention. Cronbach’s α for the total scale was 0.838, and McDonald’s *ω* was 0.842, both indicating satisfactory internal consistency (44, 45, 59).

**Table 1:** Descriptive statistics, item properties, and factor loadings from exploratory factor analysis of the GHQ-12.

Item	*M*	SD	Sk	Ku	r_it	αiid	ML1	ML2	*h* ^2^
GHQ1	1.9	0.66	−0.62	1.05	0.410	0.833	–	0.443	0.241
GHQ2	1.8	0.96	−0.26	−0.99	0.362	0.839	0.370	–	0.158
GHQ3	2.0	0.67	−0.66	1.26	0.293	0.839	–	0.418	0.179
GHQ4	2.3	0.69	−0.67	0.33	0.422	0.832	–	0.609	0.387
GHQ5	1.3	0.93	0.30	−0.75	0.476	0.829	0.611	–	0.379
GHQ6	1.7	0.83	−0.04	−0.67	0.616	0.817	0.503	–	0.427
GHQ7	2.3	0.73	−1.02	0.91	0.582	0.821	–	0.635	0.492
GHQ8	2.3	0.67	−0.73	0.95	0.510	0.826	–	0.685	0.507
GHQ9	1.5	0.93	0.11	−0.87	0.637	0.815	0.699	–	0.550
GHQ10	1.6	0.94	−0.05	−0.92	0.653	0.813	0.840	–	0.732
GHQ11	1.8	1.04	−0.31	−1.15	0.633	0.815	0.701	–	0.577
GHQ12	2.3	0.64	−0.66	0.94	0.422	0.832	–	0.519	0.298

sk, Skewness; ku, Kurtosis; r_it, Corrected item-total correlations; αiid, Cronbach’s alpha, if item deleted; *h*^2^, communality; ML1, Negative Distress; ML2, Positive Coping.

### 3.2 Exploratory factor analysis

EFA was conducted on sample 1 (*n* = 260) to explore the underlying structure of the GHQ-12. The Kaiser-Meyer-Olkin (KMO) measure was 0.858, and Bartlett’s test of sphericity was significant, *χ*^2^(66) = 994.025, *p* < 0.001, confirming the data’s factorability. The scree plot suggested a marked inflection after the second factor, which supported the two-factor solution. The two-factor solution demonstrated strong fit and interpretability, with Factor 1 (F1: Negative Distress) capturing items 2, 5, 6, 9, 10, and 11 with factor loadings ranging from 0.370 to 0.891, and Factor 2 (F2: Positive Mental Health) including items 1, 3, 4, 7, 8, and 12 with factor loadings from 0.433 to 0.711. The correlation between the two factors was moderate (*r* = 0.513), supporting the use of oblique rotation.

The two-factor structure closely corresponds to the direction of item wording (positive vs. negative). This pattern may partly reflect method effects associated with item phrasing rather than entirely distinct underlying psychological constructs (20). However, such a positive–negative wording factor structure has been consistently reported in previous GHQ-12 studies, including those conducted in Chinese populations (20, 21). Although this distinction may be useful descriptively, it should be interpreted cautiously because it may partly reflect wording-related method effects rather than fully separable substantive dimensions.

### 3.3 Confirmatory factor analysis

The EFA results from sample 1 (*n* = 260) identified a two-factor structure (Positive Mental Health and Negative Distress) with strong interpretability and balanced loadings. This empirical finding guided our decision to test the two-factor model as a core competing model in the CFA, in addition to the one-factor, three-factor, and bi-factor models specified based on theoretical and empirical literature. The two-factor model was specified exactly as suggested by the EFA solution, enabling cross-validation of the emergent structure in an independent sample.

CFA was performed on sample 2 (*n* = 251). We tested four competing models: the one-factor model (7); Two-factor model, with positive and negative wordings (21, 25, 29); Three-factor model with latent factor structure namely social dysfunction, anxiety, loss of confidence (8, 21, 31); and a bi-factor model, including a general distress factor with orthogonal positive and negative specific factors (10, 24, 26–28).

Table 2 presents the fit indices for all four models and the standardized factor loadings. The one-factor model (Model 1) had RMSEA and SRMR values over 0.08, which did not fulfill the minimum requirement for adequate fit. Both the two-factor (Model 2) and three-factor (Model 4) models demonstrated good fit, with the two-factor model performing slightly better on most indices: *χ*^2^(83.3)/53 = 1.57; CFI = 0.989; TLI = 0.986; RMSEA = 0.048; SRMR = 0.063. Although the three-factor solution met conventional fit criteria, it did not provide meaningful improvement over the two-factor model and was judged psychometrically unstable, as the ‘loss of confidence’ factor was defined by only two items (items 10 and 11); latent factors are generally expected to have at least three indicators for adequate reliability and stability (58, 61). For this reason, the three-factor model was not retained.

**Table 2:** Confirmatory factor analysis model fit indices and standardized factor loadings for the different models of GHQ-12.

Item	Model 1	Model 2		Model 3			Model 4		
	1-Factor	2-Factor		Bi-Factor			3-Factor		
	General	Positive	Negative	General	Positive	Negative	Social dysfunction	Anxiety	Loss of confidence
GHQ1	0.507	0.576	–	0.590	**0.042**	–	0.576	–	–
GHQ2	0.392	–	0.423	**0.225**	–	0.411	–	0.429	–
GHQ3	0.456	0.512	–	0.425	**0.258**	–	0.512	–	–
GHQ4	0.472	0.531	–	0.498	**0.167**	–	0.531	–	–
GHQ5	0.535	–	0.585	**0.296**	–	0.605	–	0.594	–
GHQ6	0.697	–	0.743	0.615	–	0.384	–	0.759	–
GHQ7	0.700	0.817	–	0.741	**0.250**	–	0.816	–	–
GHQ8	0.616	0.722	–	0.540	0.799	–	0.723	–	–
GHQ9	0.697	–	0.735	0.485	–	0.568	–	0.752	–
GHQ10	0.750	–	0.788	0.547	–	0.574	–	–	**0.805**
GHQ11	0.728	–	0.757	0.592	–	0.451	–	–	**0.771**
GHQ12	0.417	0.503	–	0.403	0.332	–	0.502	–	–
Model fit									
*n*	251	251		251			251		
RMSEA	**0.106**	0.048		0.026			0.049		
SRMR	**0.098**	0.063		0.048			0.063		
*χ* ^2^	205.8	83.3		48.8			81.8		
df	54	53		42			51		
*χ*^2^/df	3.81	1.57		1.16			1.60		
CFI	0.943	0.989		0.997			0.988		
TLI	0.931	0.986		0.996			0.985		

RMSEA, root mean square error of approximation; SRMR, standardized root mean residual; CFI, Comparative Fit Index; TLI, Tucker Lewis Inde, Bold values indicate specific factor loadings that failed to meet the 0.30 threshold.

The bi-factor model (Model 3) yielded the numerically best statistical fit (*χ*^2^(48.8)/42 = 1.16; CFI = 0.997; TLI = 0.996; RMSEA = 0.026; SRMR = 0.048). However, its specific factor loadings were uneven and generally weak, with many items having values below 0.30 (indicated as bold in Table 2). Specifically, for the positive specific factor, items GHQ1 (0.042), GHQ3 (0.258), GHQ4 (0.167), and GHQ7 (0.250) all fell below 0.30, while GHQ12 (0.332) just exceeded the threshold. For the negative specific factor, GHQ2 (0.411), GHQ6 (0.384), GHQ9 (0.568), GHQ10 (0.574), GHQ11 (0.451), and GHQ5 (0.605) met the 0.30 threshold, but these loadings were considerably weaker than the general factor loadings for the same items (ranging from 0.225 to 0.741). To further evaluate the dimensionality of the GHQ-12 and strengthen model comparison, bi-factor-specific variance decomposition indices were examined. The bi-factor model yielded an Omega Hierarchical (*ωh*) of 0.565 and an Explained Common Variance (ECV) of 0.565. These values indicate that the general distress factor accounted for approximately 56.5% of the reliable and common variance. While this reflects a moderately strong general factor, the relatively modest values suggest that the Positive and Negative specific factors capture meaningful unique variance, most likely attributable to item wording effects. This indicates that the Positive and Negative specific factors primarily capture residual wording/method effects after the strong general factor is accounted for. In contrast, the two-factor model produced strong, balanced loadings on all items (range = 0.423 to 0.817). The results indicated that the two-factor model (Model 2) was retained as the preferred solution, as the CFA results fulfilled all the accepted criteria for good fit, and all items’ factor loadings exceeded the 0.3 threshold. The retained two-factor solution was comparatively easier to interpret and more parsimonious, although its factors also appeared to reflect item wording direction.

### 3.4 Convergent validity

Convergent validity was examined by correlating GHQ-12 scores with the World Health Organization Well-Being Index (WHO-5) and the Short Warwick-Edinburgh Mental Well-Being Scale (SWEMWBS) in sample 2 (*n* = 251; Table 3) to preserve analytical independence. The GHQ-12 reported significant moderate negative correlations with WHO-5 and SWEMWBS, with *r* = −0.339 *(p* < 0.001) and *r* = −0.475 (*p* < 0.001), respectively. The Positive Mental Health showed moderate and significant negative correlations with the WHO-5 (*r* = −0.361, *p* < 0.001) and the SWEMWBS (*r* = −0.449, *p* < 0.001). The Negative Distress also demonstrated weak to moderate negative correlations with the WHO-5 (*r* = −0.212, *p* < 0.001) and the SWEMWBS (*r* = −0.336, *p* < 0.001).

**Table 3:** Correlations between GHQ-12 two-factor model’s latent factors with WHO-5 and SWEMWBS.

Scale	GHQ-12	GHQ-12: Positive Mental Health	GHQ-12: Negative Distress
WHO-5	−0.339	−0.361	−0.212
SWEMWBS	−0.475	−0.449	−0.336
GHQ-12	–	0.721	0.870
GHQ-12: Positive Mental Health	–	–	0.284
GHQ-12: Negative Distress	–	–	–

All correlations are significant at the 0.001 level (2-tailed).

## 4 Discussion

The present study provides a comprehensive psychometric evaluation of the General Health Questionnaire-12 in a Chinese sample, examining its reliability, factor structure, and convergent validity. Internal consistency reliability was excellent for the GHQ-12 total score with Cronbach’s alpha and McDonald’s omega well exceeding the 0.80 threshold for good reliability. Significant negative correlations between the GHQ-12 and the comparison measures also indicated good convergent validity. As such, the GHQ-12 replicated a moderate negative association with the WHO-5 (46–48) and SWEMWBS (49, 50). These findings support the GHQ-12 as a reliable and valid instrument for assessing psychological distress in this population, although convergent validity with more direct distress measures remains to be established.

The EFA yielded a two-factor model, mirroring Andrich and van Schoubroeck (25) framework of positive and negative worded structure; this structure is corroborated in prior Chinese validations, including Chinese rural residents (21) and Chinese pregnant women sample (30). The CFA revealed that the bi-factor model provided the best fit, highlighting a predominant general distress factor alongside specific factors attributable to positive- and negative-wording effects. This bi-factor preference aligns with Hystad and Johnsen (27) and Ushakova, McKenzie‘s (28) finding and Gnambs and Staufenbiel (10) meta-analytic conclusion that the general factor accounts for most variance, rendering subscales with limited unique reliability. However, in the present bi-factor solution, the general factor captured the preponderance of shared variance across items, while the positive and negative specific factors exhibited relatively weak and uneven loadings after accounting for the general factor. This pattern suggests that the specific factors primarily represent residual method effects attributable to item wording (positive vs. negative phrasing) rather than robust, substantively distinct psychological dimensions, limiting their interpretability and incremental value beyond the general factor. Although the bi-factor model showed the best numerical fit, the two-factor model was preferred on grounds of parsimony, interpretability, and the weak incremental value of the specific factors.

As reported in Model 2 (Table 2), the two-factor model demonstrated good fit (CFI = 0.989; TLI = 0.986; RMSEA = 0.048; SRMR = 0.063), with balanced loadings on theoretically meaningful dimensions: Positive Mental Health (functioning, enjoyment, and self-efficacy) and negative affect/distress (anxiety, depression, and loss of confidence). However, these two factors should be interpreted cautiously because they also closely follow positive versus negative item wording. The moderate correlation between factors (*r* = 0.616) aligns with expectations for related but distinguishable aspects of psychological wellbeing. This positive–negative distinction is one of the most consistently reported structures in the GHQ-12 literature and offers clear conceptual advantages for subscale scoring and interpretation in applied settings (21, 29, 30, 32). The three-factor model (Model 4, Table 2) performed comparably to the two-factor solution; however, the “loss of confidence” latent factor included only two items, which did not fulfil the minimum requirement of three items per factor structure.

Taken together, our findings support the characterization of the GHQ-12 as a multidimensional measure in Chinese university students. The CFA results rejected the one-factor model, indicating that the scale is not strictly unidimensional in this population. While the bi-factor model showed superior global fit, its weak specific factor loadings—particularly on the positive factor, where many items fell below the 0.30 threshold—limited its practical interpretability. Therefore, we recommend the two-factor model as the preferred structural solution, supporting the use of both the total score (for an overall indicator of psychological distress) and the two subscales (Positive Mental Health and Negative Distress) for a more comprehensive assessment. The total score remains useful for screening purposes given the strong general factor observed in the bi-factor analysis (*ωh* = 0.565; ECV = 0.565), but researchers and practitioners should be aware that the GHQ-12 is not strictly unidimensional and that subscale scores provide additional information about the nature of an individual’s psychological distress. These findings help explain the factor structure of the GHQ-12 in Chinese university students, a group facing academic and developmental stressors (4). We also addressed a gap in previous research that has primarily focused on clinical, occupational, or older adult samples (31).

### 4.1 Implications and limitation

From a practical perspective, the findings support the use of the GHQ-12 as a brief screening tool for identifying general psychological distress in university settings. Students with elevated scores may be referred for further assessment, counselling, or preventive support. The results also suggest that practitioners and researchers should be cautious when interpreting positive and negative item groupings as substantively distinct subscales, because these patterns may partly reflect wording effects rather than clearly separable clinical dimensions.

Several limitations should be acknowledged. First, the sample was drawn from a single university and was predominantly female (85.5%), which limits the generalizability of the findings to broader student populations, particularly to male university students and to institutions with different gender compositions. Although the sample reflected the gender distribution of the participating university, this demographic imbalance may have influenced the factor structure and should be addressed in future research through targeted recruitment of male participants to enable measurement invariance testing across gender (24). Second, the response rate was low (approximately 2.56%), which raises the possibility of self-selection or non-response bias. Students who chose to participate may differ systematically from non-respondents in levels of psychological distress or help-seeking attitudes, which may limit the generalizability of the findings. Third, the cross-sectional design did not allow examination of temporal stability or predictive validity. Fourth, all measures were self-reported, which may have introduced shared method variance, although Harman’s single-factor test did not provide clear evidence of a dominant single-method factor. Fifth, the external validation strategy relied on wellbeing measures rather than conceptually closer indicators such as anxiety or depression symptoms, which limits conclusions regarding convergent validity for psychological distress. Finally, measurement invariance across gender or other demographic groups was not examined, and the observed two-factor solution may partly reflect wording effects rather than fully distinct substantive dimensions.

## 5 Conclusion

In this sample of Chinese university students, the GHQ-12 demonstrated acceptable reliability and construct-related validity. Although the bi-factor model provided the strongest statistical fit, the two-factor solution was retained because it offered a more parsimonious and interpretable representation of the data. These findings support the use of the GHQ-12 as a brief distress screening measure in university populations, while also indicating that wording effects should be considered when interpreting its internal structure.

## Data Availability

The raw data supporting the conclusions of this article will be made available by the authors, without undue reservation.
